# Adults with osteogenesis imperfecta: Clinical characteristics of 151 patients with a focus on bisphosphonate use and bone density measurements

**DOI:** 10.1016/j.bonr.2018.04.009

**Published:** 2018-04-25

**Authors:** Luuk J.J. Scheres, Fleur S. van Dijk, Arjan J. Harsevoort, Atty T.H. van Dijk, Anne Marieke Dommisse, Guus J.M. Janus, Anton A.M. Franken

**Affiliations:** a Expert Center for adults with Osteogenesis Imperfecta, Isala Hospital, Zwolle, The Netherlands; b Department of Clinical Genetics, University Medical Center Groningen, Groningen, The Netherlands; c North West Thames Regional Genetics Service, Ehlers-Danlos Syndrome National Diagnostic Service London, North West Health Care University NHS Trust, Harrow, Middlesex, UK; d Expert Center for Skeletal Dysplasia, Wilhelmina Children's Hospital, University Medical Center Utrecht, The Netherlands

**Keywords:** Osteogenesis imperfecta, Adult, Expert center, Fractures, Bisphosphonates, Bone density

## Abstract

An expert center for adults with Osteogenesis Imperfecta (OI) has been founded at the Isala Hospital in Zwolle, the Netherlands to achieve optimal care for adults with OI. Clinical data such as patient history, Dual Energy X-ray Absorptiometry measurements and laboratory findings are collected with patient consent. This study provides an overview of clinical characteristics of the patients who visited the clinic during its first 5 years, a total of 151 patients. In this study, we focus on bisphosphonate use and bone density measurements at time of presentation at the expert center. As such, insight into the natural history of OI in adults will be increased. Analysing the data of a large group of adults with this rare disorder within a national expert center will allow detailed exploration of the course of OI over time.

## Highlights

• The reported cohort of 151 adult individuals with OI is one of the largest adult cohorts described in the literature.
• 45/145 patients (31%) had a 25OH-vitamin D levels <50 nm/L. Only a small group reported calcium and vitamin D use.
• Men with OI type 1 aged <50 years had significantly lower *Z*-scores of the lumbar spine compared to women.
• T- and Z-scores were lower when measured at the LS compared to PF in type 1 and 4 OI patients.
• BP use was variable; of OI type 1, 3 and 4 patients respectively 46.7%, 27.8% and 59.1% had never used BP.

## 1 Introduction

Osteogenesis Imperfecta (OI) is primarily characterized by liability to fractures, often accompanied by low bone mineral density (BMD) (Van Dijk et al., 2011). Secondary features that may be present are blue sclerae, dentinogenesis imperfecta (DI), hearing loss, ligamentous laxity and short stature. The birth prevalence of OI is estimated at 6–7 per 100,000 (Steiner et al., 2015). Approximately 90% of patients have dominant OI due to heterozygous pathogenic variants in the *COL1A1* or the *COL1A2* genes, that encode the α1-chains and α2-chain of collagen type I respectively (Sykes et al., 1990; Körkkö et al., 1998). Recently, rare recessive and X-linked variants have been reported to cause OI, the majority of which result in disturbed collagen type I biosynthesis (van Dijk and Sillence, 2014; Lindert et al., 2016).

In OI patients there are significant differences in severity (amount of bone fractures, bone deformation) and the presence of secondary clinical features. This clinical variability in OI has led to a classification in five types of OI (van Dijk and Sillence, 2014). Type 1 is the most frequent type of OI. People with OI type 1 rarely have congenital fractures but when they start to walk and consequently fall, fractures occur. They have an increased number of fractures, especially during childhood, usually without bone deformation. Blue sclerae are present and all other secondary features may also occur. OI type 2 is the perinatal lethal form with fractures showing as early as 14–16 weeks of gestation. In people with OI type 3, bone fractures are visible around 18 weeks of pregnancy, and after birth fracture frequency is very high, leading to severe deformationss of the skeleton. DI is observed in many cases. The severity in people with OI type 4 is variable. Type 4 can be progressive and deforming although typically not as deforming as in OI type 3. Type 5 is a rare subtype and characterized clinically by inability of pronation and supination due to uni-or bilateral calcification of the interosseous membrane between bones of the forearm. Furthermore, in type 5 increased callus formation is often observed (van Dijk and Sillence, 2014).

No cure for OI exists. Supportive management consists of orthopedic treatment, physical therapy, dental treatment and/or treatment for hearing loss. Calcium and vitamin-D supplements are considered useful supportive agents as they are vital in bone physiology (Clarke, 2008), and are often prescribed as they are well tolerated and inexpensive. Pharmacological treatment is also available for patients with OI, namely oral or intravenous bisphosphonates (BP). These medications inhibit bone turnover by decreasing osteoclast activity, therefore increasing overall bone mass, and providing greater skeletal strength (Glorieux et al., 1998). This treatment is often started in all types of OI patients as they frequently have significantly reduced bone density in at least one area of the skeleton.

A recent Cochrane systemic review concluded that bisphosphonates indeed increase BMD in children and adults with OI. It is still unclear whether they consistently decrease fractures, though multiple studies report this independently. The studies that were analyzed did not show that bisphosphonate treatment conclusively improves clinical status (reduce pain, improve growth and functional mobility) (Dwan et al., 2016). BP are the mainstay of care in children with moderately deforming to severe OI but may be less effective in adult patients with OI. Discussion exists as well on the long term side effects of BP's such as atypical femur fractures, osteonecrosis of the jaw (ONJ) and continuous suppression of bone turnover affecting linear growth in children (Marom et al., 2016).

The main limitation of bisphosphonate therapy is that BP are not aimed at the primary defect in OI: abnormal and/or decreased collagen type I production by osteoblasts. Other pharmacological therapies that are currently being investigated as treatment for OI include Denosumab (a RANKL inhibitor) and anabolic agents such as Teriparatide (recombinant form of parathyroid hormone), Cathepsin K inhibition, Growth hormone, Sclerostin-inhibitory antibodies and TGFβ inhibition (Marom et al., 2016).

To achieve optimal care for adult people with OI and increase insight into all aspects of this disorder, an expert center for adults with OI was founded in the Netherlands with strong support of the Dutch OI patient organization. Clinical data obtained as part of routine care, such as patient history, BMD measurements and laboratory findings have been stored, with patient consent, into an anonymized database. This study reports the clinical characteristics of 151 adult patients with OI with a focus on both current as previous bisphosphonate use and bone density measurements at time of visit.

## 2 Materials and methods

The results of this study are derived from the data of a large group of adults with OI that have been seen in clinic by the multidisciplinary team from the Isala Teaching Hospital, Zwolle, the largest non-university hospital in the Netherlands. In one day, patients are seen separately by members of the multidisciplinary team: the coordinator (an advanced nurse practitioner), orthopedic surgeon, internist-endocrinologist, rehabilitation physician, occupational therapist and clinical geneticist. Informed consent was obtained from the patients to retrieve and store clinical, radiological and laboratory data in a database. Data from the first visits of 151 patients ≥18 years with a clinically confirmed diagnosis of OI were included in this study. Data presented in this study are obtained from medical history as reported by the patient and regarding bisphosphonate use (which of the BP were used, time course of treatment, which dose of BP was used, and mode of administration) medical correspondence was also analyzed to confirm whenever possible. Furthermore, data were obtained based on physical (orthopedic) examination (height and weight are measured with standardized equipment), laboratory tests, radiographs, and DXA-scans.

### 2.1 Bone mineral density

Bone mineral density (BMD) was assessed by means of a DXA-scan (Discovery-A, Hologic). All analyses were performed at our clinic and the same scanner was used. BMD was measured at the lumbar spine (LS) L1 to L4 and at the proximal femur (PF) of the left leg. In an age-matched normal population sample, the coefficients of variation (CV) were 0.669% at the LS and 1.0% at the femoral neck. Data from severely deformed bones or implants in the measured region were excluded. When assessment of the left PF was not possible, the BMD at the right PF was measured, if possible. BMD is expressed in T (age specific)- and Z (age and sex-specific)-scores. To gain insight in the clinical status of the patient, BMD was categorized according to the International Society for Clinical Densitometry (Schousboe et al., 2013) and subsequently the WHO-osteoporosis classification (Organization WH, 1994).

### 2.2 Laboratory tests

The laboratory tests that were performed were part of routine care and were done by using standard laboratory techniques and included determination of: erythrocyte sedimentation rate, hemoglobin, leukocytes, creatinine, calcium, phosphate, alkaline phosphatase, y-glutamyl transferase, albumin, c-reactive protein, free T4, thyroid stimulating hormone, parathyroid hormone, telepeptide-C and 25-OH vitamin D levels.

### 2.3 Statistical analysis

Clinical characteristics and biochemical measurements are presented as the mean ± the standard deviation (SD) or as proportions (%). To investigate the difference in measured BMD (for T-scores and *Z*-scores separately) per OI type at the lumbar spine and proximal femur in individual patients, a paired *t*-test was used. The mean differences are presented as the mean with 95% confidence intervals (95%CI). To explore a difference in BMD among males and females, per OI type, an independent *t*-test was conducted, and mean differences are presented with 95%CI.

## 3 Results

### 3.1 Overview of clinical characteristics

Clinical characteristics of the study cohort are shown in Table 1. Most patients (*n* = 107) have been diagnosed with OI type 1, and most patients were female (*n* = 100). In type 1, 30/107 (28%) were male, in type 3 9/21 (43%) were male and 12/23 (52%) type 4 patients were male. The median age was 41 years for type 1 patients, 26 years for type 3 and 31 years for type 4. The oldest patients were 76 for type 1, 62 for type 3 and 63 years for type 4. Type 1 patients had a median height of 164 cm, this was 105 and 155 cm for type 3 and 4 respectively. Independent of age, most type 1 patients had sustained 0–15 fractures at time of presentation, the majority of type 3 patients had >45 fractures when visiting our clinic. The amount of fractures sustained was more variable in type 4 patients: 39.2% had 0–15 fracture, whereas 26.1% had 36 or more fractures. Of the patients for whom data were available, all with type 1 had blue sclera. Dentinogenesis imperfecta was the most common among type 3 (67%) patients, followed by type 4 (31%), and was observed in 21% of those type 1 patients where this information was available.

**Table 1:** Overview of clinical characteristics of the study cohort.

OI type		1	3	4
Individuals (n)		107	21	23
Gender (Male;Female)		30;77	9;12	12;11
Age (years)		41	26	31
Median, (min–max)		(18–76)	(18–62)	(18–63)
Height (cm)		164	105	155
Median, (min–max)		(126–190)	(83–150)	(115–181)
Total suffered fractures at the time of first visit (%)	0–15 (fractures)	59.0%	10.5%	39.2%
	16–25	17.1%	5.3%	21.7%
	26–35	9.5%	5.3%	13%
	36–45	4.9%	10.5%	8.7%
	>45	9.5%	68.4%	17.4%
Surgery for at least 1 fracture (%)		83/91 91.2%	90%	94.4%
Wheelchair users (%)		13.1%	100%	39.1%
Blue Sclera, n (%)		53/53^a^ (100%)	3/8^a^ (37.5%)	4/10^a^ (40%)
Dentinogenesis Imperfecta, n (%)		10/47^a^ (21%)	6/9^a^ (67%)	4/13^a^ (31%)
BMD proximal femur	T-score, mean ± SD	−1.29 ± 1.20	−2.82 ± 2.17	−1.18 ± 1.58
	*Z*-score, mean ± SD	−1.01 ± 1.13	−2.30 ± 1.97	−1.32 ± 1.23
BMD lumbar spine	T-score, mean ± SD	−2.46 ± 1.23	−4.48 ± 0.81	−2.44 ± 1.73
	*Z*-score, mean ± SD	−2.00 ± 1.27	−4.08 ± 1.07	−2.30 ± 1.51
Bisphosphonate treatment never, n (%)		49/106 (46.2%)	5/18 (27.8%)	13/22 (59.1%)
Calcium/Vit. D3 supplements, n (%)		18/90^a^ (20%)	3/15^a^ (20%)	3/19^a^ (16%)

a The characteristic was analyzed in the patients of which these characteristics were available.

### 3.2 BMD measurements

Table 2 shows the *Z*-scores of patients <50 years and the T-scores for patients ≥50 years, per region of measurement according to the International Society for Clinical Densitometry. Independent of BP use, for type 1 patients the average *Z*-score (±SD) for the LS was −1.77 ± 1.08 for females aged <50 compared with −2.86 ± 1.37 for males aged <50 (mean difference 1.08, 95% CI 0.38 to 1.78). For the Z-score of the PF this was −0.93 ± 0.91 for the females aged <50 and −1.55 ± 1.53 for the males <50 (mean difference 0.61, 95%CI −0.17 to 1.21). For type 1 females aged ≥50 the average Z-score at the LS was −1.63 ± 1.2, and for the PF -0.84 ± 1.01. This was −2.12 ± 1.47 at the LS (mean difference 0.49, 95%CI −0.54 to 1.52) and − 0.55 ± 1.25 at the PF (mean difference − 0.29, 95%CI −1.2 to 0.62) for males with type 1 aged ≥50.

**Table 2:** T- and *Z*-scores according to the International Society for Clinical Densitometry [25] (with Z-scores for patients <50 years of age and T-scores for patients ≥50 years of age).

OI type	1			3			4		
DXA location	LS	PF	OTHER	LS	PF	OTHER	LS	PF	OTHER
Patients <50 years of age, Z-scores									
Z- score >−2.0	52.2% (*n* = 36)	82.4% (*n* = 56)	80% (*n* = 8)	–	50% (*n* = 2)	–	35.7% (n = 5)	61.5% (n = 8)	40% (n = 2)
Z-score ≤−2.0 below expected range for age	47.8% (*n* = 33)	17.6% (*n* = 12)	20% (n = 2)	100% (n = 3)	50% (n = 2)	100% (*n* = 6)	64.3% (*n* = 9)	38.5% (n = 5)	60% (n = 3)

Patients ≥50 years of age, T-scores (according to WHO classification [26])									
−1.0 or higher	3.1% (n = 1)	26.7% (n = 8)	50% (n = 1)	–	100% (n = 1)	–	–	16.7% (n = 1)	50% (n = 1)
>−2.5 to <−1.0	37.5% (*n* = 12)	56.6% (*n* = 17)	50% (n = 1)	–	–	50% (n = 1)	16.7% (n = 1)	66.6% (n = 4)	–
≤−2.5	59.4% (*n* = 19)	16.7% (n = 5)	–	100% (n = 1)	–	50% (n = 1)	83.3% (n = 5)	16.7% (n = 1)	50% (n = 1)

LS = Lumbar Spine, PF = proximal femur, OTHER = Distal radius and ulna or the wrist.

For type 1 patients, the mean T-score of the LS was 1.15 lower (95%CI −1.38 to −0.91, *p* ≤0.001) compared to the T-score at the PF, in the same patient at the same time. This was −1.35 lower at the LS compared to the PF for type 4 patients (95%CI −2.35 to −0.35, *p* = 0.011). This difference in the *Z*-score of the LS and the PF was on average − 0.96 (95%CI −1.20 to −0.72, p ≤0.001) for type 1 patients. For type 4 the mean difference between the *Z*-score of the LS was −0.88 (95%CI −1.49 to −0.27, *p* = 0.007) compared with the PF measurement. As data on BMD of only few type 3 patients were available, formal statistical testing was not performed. Two type 3 patients <50 years had normal BMD measurements at the PF, this was the case for 1 patient ≥50 years.

### 3.3 Bisphosphonates

About half of type 1 (467%) and 4 (59.1%) patients had never been treated with BP. However, only a minority of type 3 (27.8%) patients had not had BP treatment. Type 1 patients in particular (31.4%) were treated for longer than 5 years in adulthood. Of the type 3 patients who had ever used BP, 27.8% reported having been treated both as a child as in adulthood. Few patients (2.9% for type 1, none in type 3 and 9.1% for type 4) reported treatment in childhood only (Table 3).

**Table 3:** Bisphosphonate use per OI type.

BP use	1 (*n* = 105)	3 (*n* = 18)	4 (*n* = 22)
Never	46.7%	27.8%	59.1%
Childhood only	2.9%	–	9.1%
Adulthood <5 years	10.5%	27.8%	9.1%
Adulthood ≥5 years	3144%	16.6%	18.1%
Childhood and adulthood	8.5%	27.8%	4.6%

Five patients with OI type 3 reported having never used BP. Table 4 shows the different BP types used by patients. Risedronate accounted for most of all prescribed BP among all patients. Alendronate was used by over a quarter of the remaining patients. A smaller fraction of patients was prescribed Pamidronate, or Ibandronate. Type 3 patients had been treated with more than one different type of BP at different times periods.

**Table 4:** Bisphosphonate type used per OI type.

BP types/ OI type	1 (*n* = 52)	3 (*n* = 13)	4 (n = 8)
Risedronate	42.4%	46.1%	75%
Alendronate	32.6%	15.4%	12.5%
Pamidronate	5.8%	7.7%	12.5%
Alendronate + calciferol	5.8%	7.7%	–
Ibandronate	1.9%	–	–
Zoledronate	1.9%	–	–
Combination of BP^a^	9.6%	23.1%	–

a Different types of BP were used, at different time periods.

At time of presentation at our expert center, one patient with OI type 1 had a history of osteonecrosis of the jaw, this patient reported having used BP for over 5 years.

### 3.4 Biochemical parameters

Table 5 shows the mean values of laboratory findings and occurrence of values outside of the reference range for OI patients per type at time of presenting at our clinic. About 30% of all patients (*n* = 45) (a quarter of all type 1 patients, and almost half of the patients with type 3 and 4 of whom laboratory measurements were available), had 25OH-vitamin D levels <50 nm/L. Of these, 30% (*n* = 14) had an elevated parathyroid hormone (PTH) level secondary to a low vitamin D levels. Other laboratory tests were in most cases within normal range or only slightly outside of reference ranges. In a few patients, minor abnormalities were present among the laboratory findings, however these were not apparently associated with OI.

**Table 5:** Mean and occurrence of abnormal values of biochemical parameters for adults with osteogenesis imperfecta per type.

Type	1	3	4
Number of participants*	101	21	23
25OH-vitamin D nmol/L, mean (±SD)	71.9 (31.8)	56.6 (29.9)	59.8 (33.2)
25OH-vitamin D < 50 nmol/L	24/101 (23.8%)	10/21 (47.6%)	11/23 (47.8%)
Parathyroid hormone pmol/L, mean (±SD)	4.3 (1.6)	4.5 (2.0)	4.2 (1.7)
Parathyroid hormone >7 pmol/L	10/101 (9.9%)	3/21 (13.3%)	1/23 (4.3%)
Calcium, mean (±SD)	2.3 (0.1)	2.4 (0.1)	2.4 (0.1)
Calcium, <2.10 or > 2.55	2/101 (2.0%)	0/21 (0%)	1/23 (4.3%)
Phosphate mmol/L, mean (±SD)	1.0 (0.2)	1.1 (0.2)	2.4 (0.1)
Phosphate, <0.8 or > 1.5 mmol/L	23/101 (22.8%)	1/21 (4.8%)	11/23 (47.8%)
Alkaline phosphatase mmol/L, mean (±SD)	77.6 (27.4)	88.0 (22.8)	92.8 (54.8)
Alkaline phosphate >125 mmol/L	15/101 (14.9%)	2/21 (10.0%)	9/23 (39.1%)
Telopetide-C pg/ml, mean (±SD)	183.7 (117.3)	215.7 (209.5)	232.5 (140.6)
Telopetide (*Z*-score), mean (±SD)	0.7 (12.0)	−0.7 (1.3)	−0.4 (0.7)

For several patients, laboratory measurements or values were missing (Supplementary Table 1).

For several patients, laboratory measurements or values were missing (Supplementary Table 1).

## 4 Discussion

In this study we describe the clinical characteristics of a large cohort of 151 Dutch adults with OI. To our knowledge, this is one of the largest adult cohorts described in the literature. Wekre and colleagues (Wekre et al., 2011), reported in 2011 on 97 adults with OI with a focus on bone mass, bone markers and fracture prevalence. Lindahl and colleagues (Lindahl et al., 2015) described detailed clinical phenotypes of a cohort of 223 OI patients, including 119 adults. Recently, Hald and colleagues (Hald et al., 2016) reported on 85 adult OI patients. These studies focused on genotype-phenotype correlations. In our adult cohort we observed a female predominance in type 1 patients, although type 3 and 4 were smaller patient groups. In the adult population reported by Wekre et al. a female majority was seen in the OI type 1 and type 4 (Wekre et al., 2011). Lindahl et al. noted that the gender distribution was slightly skewed for OI type 3 (F > M), probably due to the small study group size (Lindahl et al., 2015). Hald et al. observed a female majority only in OI type 1 (Hald et al., 2016). The height of type 1 patients approximated the average height of the Dutch general population, which is 173.9 cm (Statistics Netherlands, 2015), especially when taking a female predominance among type 1 patients in this into account. The median height of type 3 and 4 patients was substantially lower than the Dutch average. Independent of age and sex, the total number of fractures at time of presentation at the expert center varied among the patients, with most fractures in type 3 patients, followed by type 4 patients and the fewest fractures in type 1 patients. The same observation was made by Lindahl and colleagues, with on average lowest amount of fractures in type 1 adults, and most observed in type 3 adults. Hald and colleagues noticed a comparable average amount of fractures in adults with type 1 and type 4 OI and most fractures were observed in adults with OI type 3 as well (Hald et al., 2016).

A considerable number of patients 45/145 (31%) had a 25OH-vitamin D levels <50 nm/L at their first visit of our out-patient clinic. Only a small group reported consuming calcium and vitamin D supplements. Wekre et al. reported use of calcium and vitamin D in 25% of all OI patients (Wekre et al., 2011). This was certainly different in the adults reported by Hald et al. where 65% received supplementation with calcium and vitamin D (Hald et al., 2016). One could argue that perhaps in all OI patients, regardless of BMD or fracture frequency, normal vitamin D levels are important, as vitamin D plays a key role in normal bone metabolism. Most other laboratory findings were within normal limits, as is expected as serum calcium values have been reported to be generally within normal ranges in patients with OI and previous reports mention that bone turnover markers can be decreased, increased or normal (Wekre et al., 2011).

Measurement of BMD by means of DXA has proven to be a useful instrument to evaluate disease progress and/or treatment effect in individual patients. Regarding bone density measured by DXA, many patients have a normal BMD according to the classification of the International Society for Clinical Densitometry. On average, type 1 males aged <50 had significantly lower *Z*-scores of the lumbar spine compared with type 1 females <50, an observation also described by Lindahl et al. (Lindahl et al., 2015). Hald et al. showed that aerial BMD (aBMD) was significantly lower in patients with OI type 3 compared to patients with OI type 4 (*p* < 0.0001). However, aBMD did not differ significantly between patients with OI type 1 and 4 (Hald et al., 2016). This finding was replicated in the group of OI patients reported here. Wekre et al. found significant differences between patients with OI type 3 and type 1 and 4 with respect to total body BMD (*p* < 0.001) and total body BMD Z score (*p* = 0.02) (Wekre et al., 2011).

In our patient group of OI patients, both T and Z scores in the same patient at the same time were considerably lower when measured at the LS compared with the measurement of the PF in type 1 and 4 patients. Discrepancy of DXA results among different regions of the body in adult OI was documented before (Seeman and Delmas, 2006), and our results add to these previous reports. An explanation for this finding could be difference in make-up of bone tissue at the different sites. The LS is enriched with trabecular bone opposed to the PF which is mainly made up of cortical bone. These differences could explain the differences in measured BMD. When evaluating BMD of OI patients over time, this discrepancy between body regions should be taken into account. Three type 3 patients had normal measurements at the PF, emphasizing that DXA does not grant insight in the composition or structural design of the bone (Seeman and Delmas, 2006). Hald et al. reported on the use of High-resolution peripheral quantitative computed tomography (HRpQCT), to assess bone geometry, volumetric BMD (vBMD), and micro architecture with scans performed on the non-dominant radius and tibia. Interestingly, they noticed that patients with mild OI type 1 have more severely impaired bone structure and microarchitecture, as assessed by HRpQCT, when compared to patients with OI type 4 (Hald et al., 2016).

In this study the current and previous use of BP was assessed in contrast to other studies which only reported on current use. Wekre et al. noted that 18% of all OI patients were using BP and/or hormone replacement therapy (Wekre et al., 2011). Hald et al. report that two thirds of the patients were current users of bone protective treatment without difference between the groups. This included intravenous bisphosphonates, oral bisphosphonates less frequently, and parathyroid hormone received by two patients (Hald et al., 2016). The current and previous use of BP varies in our group of patients. Decisions to start BP treatment in current practice will mainly depend on measured BMD and fracture frequency together with the tolerance of the treatment by patients. Effectiveness of BP in terms of fracture reduction has been demonstrated in several studies in children with BP (Organization WH, 1994). BP use seems to increase the BMD in adults with OI, however the effectiveness on fracture reduction in adults remains uncertain (Wekre et al., 2011; Lindahl et al., 2015; Hald et al., 2016). About half of all type 1 and 4 patients reported never having used BP in their life, although nowadays early treatment in childhood is recommended. As no data on their childhood treatment was available it is uncertain why no treatment was prescribed at that time. It is possible that these patients had low fracture rates during childhood or were diagnosed with OI after childhood. In the entire cohort, only a few patients had used BP in childhood, although the evidence of effectiveness of BP is strongest in children. One explanation is that in this adult cohort many patients had already reached adulthood before the availability of BP. Interestingly, three type 3 patients reported no history of BP use. Although one could argue that BP treatment would be essential in the most severe nonlethal OI type, preventive measures such as fall and trauma prevention are the most important in preventing fractures in OI type 3. The additive effect of BP to these preventive measures in fracture reduction in type 3 patients has not been studied extensively.

## 5 Conclusion

By describing clinical data of a large group of adults with OI a clinical picture of the adult patient with OI type 1, 3, 4 emerges. The national expert center for adults with OI allows recorded follow up from initial consultation into a single database, which in turn will allow exploration of many important aspects and questions in the future regarding Osteogenesis Imperfecta over time. A few examples are: the efficacy of BP treatment in adults with OI, occurrence of cardiovascular complications, occurrence of respiratory complications and genotype-phenotype studies. As more knowledge about adults with OI becomes available, current outcome measures for successful treatment of OI such as fracture incidence need to be reviewed.

The following is the supplementary data related to this article.

Supplementary Table 1 All data of laboratory measurements.

## Transparency document

Transparency document Image 1
