# Supplementary material for: Adults with osteogenesis imperfecta: Clinical characteristics of 151 patients with a focus on bisphosphonate use and bone density measurements
Source: Bone Rep. 2018 Apr 25;8:168–72. doi: 10.1016/j.bonr.2018.04.009 (PMC6020398; doi:10.1016/j.bonr.2018.04.009)
Supplement: Supplementary Table 1 [file mmc1.docx]

**Supplemental data**

**Supplemental table 1.**

| # | OI Type | Telopeptide-C (pg/ml), normal range*:  80-680 pg/ml | Telopeptide (Z-score)  normal range: | Calcium (mmol/L)  normal range: 2,10-2,55 | 25 OH-Vitamin D (nmol/L)  normal range: 50-150 | PTH (pmol/L)  normal range:  <7pmol/l | Phosphate (mmol/L)  normal range:  0.8-1.5 | AF (mmol/L)  normal range:  <105U/L |
| --- | --- | --- | --- | --- | --- | --- | --- | --- |
| 1 | 4 | - | - | 2.33 | 93 | 2 | 0.86 | 78 |
| 2 | 4 | 129 | -0.97 | 2.63 | 95 | 3.9 | 1.45 | 47 |
| 3 | 1 | 28 | -1.42 | 2.16 | 79 | 2.3 | 0.75 | 56 |
| 4 | 1 | 102 | -1.1 | 2.35 | 66 | 3.3 | 1.11 | 86 |
| 5 | 4 | 44 | -1.4 | 2.55 | 78 | 2 | 0.82 | 83 |
| 6 | 1 | 373 | 1.68 | 2.34 | 69 | 3.7 | 0.99 | 97 |
| 7 | 1 | 71 | -1.42 | 2.24 | 95 | 4.8 | 0.96 | 65 |
| 8 | 4 | 359 | 0.46 | 2.22 | 55 | 3.9 | - | - |
| 9 | 1 | 161 | -0.38 | 2.37 | 78 | 5.1 | 1.09 | 90 |
| 10 | 1 | 257 | -0.92 | 2.31 | 36 | 3.5 | 0.84 | 102 |
| 11 | 1 | 237 | 0.34 | 2.15 | 58 | 5.4 | 1.04 | 80 |
| 12 | 1 | 250 | -0.44 | 2.22 | 42 | 4 | 0.95 | 46 |
| 13 | 3 | 119 | -1.14 | 2.19 | 35 | 8.6 | 1.14 | 110 |
| 14 | 4 | 213 | -0.45 | 2.29 | 110 | 3.5 | - | - |
| 15 | 1 | - | - | - | - | - | - | - |
| 16 | 1 | 116 | -1.08 | 2.3 | 31 | 3.2 | 1.34 | 61 |
| 17 | 1 | 145 | -1.13 | 2.32 | 36 | 3.9 | 0.62 | 151 |
| 18 | 1 | 223 | -0.31 | 2.23 | 121 | 5.4 | 1.16 | 73 |
| 19 | 1 | 243 | -0.06 | 2.31 | 36 | 4.2 | 1.23 | 69 |
| 20 | 1 | 51 | -1.46 | 2.46 | 112 | 3.8 | 1.08 | 59 |
| 21 | 1 | 24 | -2.02 | 2.26 | 138 | 3.6 | 1.3 | 55 |
| 22 | 1 | 362 | 0.08 | 2.21 | 50 | 3.7 | 1.32 | 62 |
| 23 | 1 | 117 | -1.08 | 2.29 | 48 | 5.2 | 1.08 | 60 |
| 24 | 1 | 76 | -1.65 | 2.23 | 114 | 6.3 | 0.9 | 84 |
| 25 | 1 | 216 | 0.33 | 2.29 | 45 | 3.9 | - | - |
| 26 | 1 | 203 | -0.02 | 2.28 | 29 | 7.4 | 0.84 | 118 |
| 27 | 1 | 134 | -0.65 | 2.24 | 25 | 8.2 | 0.84 | 132 |
| 28 | 1 | 248 | -0.46 | 2.37 | 72 | 2.9 | 0.79 | 79 |
| 29 | 1 | 251 | -0.15 | 2.26 | 57 | 4.1 | 1.09 | 59 |
| 30 | 1 | 191 | -0.26 | 2.26 | 31 | 3.7 | 1.07 | 41 |
| 31 | 1 | 66 | -1.1 | 2.33 | 58 | 5.8 | 1.36 | 63 |
| 32 | 1 | 132 | -0.77 | 2.31 | 74 | 5.2 | 0.72 | 78 |
| 33 | 4 | 137 | -1.3 | 2.31 | 41 | 3.2 | 1.16 | 51 |
| 34 | 1 | 158 | -0.88 | 2.15 | 111 | 3.2 | - | - |
| 35 | 1 | 229 | -0.81 | 2.27 | 62 | 3.3 | 0.9 | 73 |
| 36 | 1 | 101 | -0.74 | 2.32 | 37 | 3.7 | 1.17 | 82 |
| 37 | 1 | 107 | -1.23 | 2.32 | 59 | 5.1 | 1.02 | 68 |
| 38 | 1 | 308 | -0.5 | 2.43 | 36 | 4 | 1 | 96 |
| 39 | 3 | 277 | -0.55 | 2.49 | 92 | 2.7 | 1.02 | 127 |
| 40 | 1 | 621 | 1.3 | 2.29 | 56 | 3.5 | 1.08 | 146 |
| 41 | 1 | 183 | -1.1 | 2.31 | 115 | 3.4 | 1.13 | 87 |
| 42 | 1 | - | - | 2.36 | 138 | 6.5 | 0.77 | 66 |
| 43 | 1 | 98 | -1.16 | 2.37 | 130 | 2.3 | 0.56 | 44 |
| 44 | 1 | 446 | 0.95 | 2.3 | 67 | 6.4 | 0.87 | 120 |
| 45 | 1 | 431 | -0.37 | 2.41 | 62 | 4.1 | 1.3 | 53 |
| 46 | 1 | 62 | -1.14 | 2.28 | 128 | 4.2 | 1.1 | 45 |
| 47 | 1 | 163 | -0.85 | 2.25 | 127 | 2 | 1.21 | 83 |
| 48 | 4 | 228 | -0.55 | 2.46 | 30 | 2.9 | 0.82 | 81 |
| 49 | 1 | 256 | -0.32 | 2.11 | 28 | 8 | 1.02 | 47 |
| 50 | 3 | - | - | 2.37 | 16 | 4.4 | 1.31 | 107 |
| 51 | 1 | 393 | 0.43 | 2.48 | 67 | 2.9 | 1.05 | 91 |
| 52 | 1 | 102 | -1.14 | 2.54 | 97 | 1.9 | 0.72 | 53 |
| 53 | 1 | 83 | -1 | 2.35 | 79 | 2.9 | 0.83 | 56 |
| 54 | 1 | 235 | -0.43 | 2.48 | 67 | 2 | 0.65 | 81 |
| 55 | 1 | - | - | - | - | - | - | - |
| 56 | 1 | 284 | 1.31 | 2.41 | 118 | 4.9 | 1.16 | 135 |
| 57 | 1 | 20 | -2.37 | 2.21 | 57 | 3.6 | 1.05 | 47 |
| 58 | 1 | 208 | 0.07 | 2.33 | 154 | 7.6 | 0.92 | 107 |
| 59 | 3 | 180 | -1.09 | 2.51 | 23 | 2.7 | 1.14 | 88 |
| 60 | 4 | 166 | -0.84 | 2.39 | 63 | 3.3 | 0.76 | 36 |
| 61 | 1 | 445 | 0.47 | 2.47 | 20 | 3.1 | 1.2 | 77 |
| 62 | 4 | 243 | 0.61 | 2.39 | 21 | 7 | 0.81 | 96 |
| 63 | 1 | 144 | 0.59 | 2.23 | 88 | 6.1 | 0.89 | 38 |
| 64 | 4 | 129 | -0.69 | 2.37 | 14 | 6.2 | 0.65 | 61 |
| 65 | 1 | - | - | - | - | - | - | - |
| 66 | 3 | 102 | -1.59 | 2.46 | 79 | 2.8 | 0.85 | 85 |
| 67 | 4 | 242 | -0.74 | 2.29 | 39 | 3.6 | - | - |
| 68 | 1 | 209 | -0.38 | 2.57 | 89 | - | - | - |
| 69 | 1 | 250 | 0.09 | 2.33 | 20 | - | - | - |
| 70 | 1 | - | - | - | - | - | - | - |
| 71 | 1 | 250 | -0.81 | 2.43 | 73 | 4.5 | - | - |
| 72 | 4 | 219 | 0.37 | 2.33 | 94 | 3.4 | - | - |
| 73 | 3 | - | - | 2.39 | 41 | 3.7 | 1.13 | 73 |
| 74 | 1 | 246 | -0.26 | 2.32 | 61 | 2.1 | - | - |
| 75 | 1 | - | 108 | 2.36 | 100 | 1.5 | - | - |
| 76 | 4 | 248 | -0.24 | 2.36 | 41 | 4.8 | - | - |
| 77 | 4 | 56 | -1.67 | 2.31 | 111 | 5.6 | - | - |
| 78 | 1 | - | - | 2.29 | 134 | 4.3 | 0.73 | 66 |
| 79 | 3 | - | - | 2.33 | 129 | 5 | 0.83 | 99 |
| 80 | 1 | 154 | -1.22 | 2.35 | 84 | 2.3 | - | - |
| 81 | 1 | - | - | 2.36 | 84 | 3.5 | 0.85 | 45 |
| 82 | 1 | - | - | 2.41 | 70 | 3.8 | 0.88 | 74 |
| 83 | 1 | - | - | 2.48 | 78 | 3.2 | 1.04 | 77 |
| 84 | 4 | - | - | 2.55 | 45 | 3.3 | 1.06 | 69 |
| 85 | 1 | - | - | 2.33 | 140 | 9.3 | 1.2 | 58 |
| 86 | 3 | - | - | 2.53 | 76 | 5.7 | 1.19 | 54 |
| 87 | 4 | - | - | 2.36 | 95 | 3.1 | 0.77 | 47 |
| 88 | 3 | - | - | 2.4 | 33 | 3.5 | 1.26 | 127 |
| 89 | 1 | - | - | 2.3 | 75 | 4.7 | 1.13 | 99 |
| 90 | 1 | - | - | 2.29 | 10 | 8.7 | 0.83 | 75 |
| 91 | 1 | - | - | 2.39 | 47 | 6 | 1.12 | 49 |
| 92 | 1 | - | - | 2.48 | 69 | 1.9 | 1.3 | 71 |
| 93 | 1 | - | - | 2.38 | 38 | 5.8 | 0.52 | 109 |
| 94 | 1 | - | - | 2.18 | 89 | 4.6 | 1.14 | 59 |
| 95 | 4 | - | - | 2.39 | 105 | 4 | 0.99 | 115 |
| 96 | 4 | - | - | 2.43 | 59 | 5.2 | 1 | 76 |
| 97 | 3 | - | - | 2.3 | 58 | 3.9 | 0.97 | 64 |
| 98 | 4 | - | - | 2.37 | 30 | 9.1 | 0.71 | 225 |
| 99 | 1 | - | - | - | - | - | - | - |
| 100 | 1 | - | - | - | - | - | - | - |
| 101 | 1 | 297 | -0.61 | 2.38 | 78 | 1.5 | - | - |
| 102 | 1 | - | - | 2.24 | 77 | 3.9 | 0.91 | 55 |
| 103 | 3 | - | - | 2.29 | 33 | 5.3 | 1.02 | 66 |
| 104 | 1 | 311 | 0.38 | 2.23 | 56 | 6.9 | 0.9 | 62 |
| 105 | 1 | - | - | 2.34 | 59 | 4 | 0.84 | 39 |
| 106 | 1 | 143 | -1.28 | 2.22 | 65 | 5.3 | 0.91 | 122 |
| 107 | 1 | 58 | -1.13 | 2.32 | 60 | 4.4 | 0.96 | 123 |
| 108 | 3 | 74 | -1.27 | 2.48 | 53 | 2.3 |  |  |
| 109 | 1 | - | - | 2.24 | 104 | 4.2 | 0.98 | 75 |
| 110 | 1 | 73 | -1.16 | - | - | - | - | - |
| 111 | 4 | 172 | -0.28 | 2.3 | 90 | 2.5 | 0.66 | 57 |
| 112 | 3 | 199 | -0.69 | 2.42 | 49 | 5 | 1.15 | 81 |
| 113 | 3 | 130 | -0.79 | 2.31 | 94 | 3.9 | 1.01 | 88 |
| 114 | 1 | 243 | 0.34 | 2.26 | 33 | 6 | 0.84 | 80 |
| 115 | 3 | - | - | 2.44 | 74 | 3.3 | 1.29 | 79 |
| 116 | 1 | 134 | -0.35 | 2.29 | 81 | 4 | 1.01 | 90 |
| 117 | 1 | 129 | -1.01 | 2.2 | 110 | 2.3 | 0.73 | 71 |
| 118 | 1 | 27 | -1.69 | 2.33 | 91 | 4.6 | 1.1 | 40 |
| 119 | 1 | 223 | -0.21 | 2.22 | 68 | 5.5 | 0.73 | 85 |
| 120 | 1 | 120 | -1.06 | 2.19 | 69 | 5.2 | 0.86 | 95 |
| 121 | 3 | 243 | 0.67 | 2.3 | 22 | 9.2 | 1.01 | 99 |
| 122 | 3 | 218 | -0.95 | 2.18 | 18 | 5.1 | 1.14 | 123 |
| 123 | 1 | 156 | -0.43 | 2.28 | 59 | 4.1 | 0.97 | 85 |
| 124 | 1 | 50 | -1.95 | 2.18 | 73 | 3.8 | 1.05 | 92 |
| 125 | 1 | - | - | 2.39 | 20 | 7.6 | 1 | 92 |
| 126 | 3 | - | - | 2.47 | 75 | 2.2 | 1.25 | 86 |
| 127 | 1 | 206 | 0.21 | 2.35 | 65 | 5.5 | 0.88 | 98 |
| 128 | 4 | 590 | 0.14 | 2.35 | 28 | 5.8 | 0.72 | 209 |
| 129 | 3 | - | - | 2.37 | 84 | 3.6 | 1.01 | 73 |
| 130 | 1 | - | - | 2.28 | 29 | 3.5 | 1.18 | 71 |
| 131 | 1 | 52 | -1.95 | 2.26 | 118 | 3.2 | 0.8 | 82 |
| 132 | 1 | 207 | 0.06 | 2.33 | 54 | 4.5 | 1.4 | 87 |
| 133 | 1 | 136 | -0.67 | 2.25 | 56 | 5 | 1.27 | 121 |
| 134 | 1 | 42 | -1.35 | 2.13 | 46 | 6 | 1.01 | 15 |
| 135 | 1 | - | - | 2.51 | 91 | 3.3 | 1.16 | 74 |
| 136 | 4 | 482 | 0.92 | 2.39 | 20 | 4 | 0.99 | 92 |
| 137 | 3 | 805 | 2.38 | 2.36 | 64 | 3.6 | 1.14 | 47 |
| 138 | 3 | 26 | -2.17 | 2.43 | 41 | 7.6 | 1.42 | 84 |
| 139 | 1 | 10 | -2.7 | 2.33 | 87 | 2.3 | 1.14 | 37 |
| 140 | 1 | 178 | -1.17 | 2.35 | 81 | 2.8 | 0.64 | 99 |
| 141 | 1 | 119 | -0.56 | 2.11 | 75 | 4 | 1.21 | 78 |
| 142 | 1 | 155 | -0.64 | 2.34 | 81 | 3.4 | 0.81 | 86 |
| 143 | 1 | 183 | 0.21 | 2.12 | 71 | 3.2 | 1.06 | 50 |
| 144 | 1 | - | - | 2.48 | 126 | 2.1 | 0.9 | 47 |
| 145 | 1 | 210 | -0.98 | 2.24 | 90 | 3.1 | 1.11 | 62 |
| 146 | 1 | 142 | -1.28 | 2.27 | 61 | 3 | 0.95 | 99 |
| 147 | 1 | 24 | -1.95 | 2.3 | 36 | 4.8 | 0.88 | 79 |
| 148 | 1 | 279 | 0.77 | 2.38 | 55 | 5.8 | 1.29 | 117 |
| 149 | 4 | 295 | -0.8 | 2.23 | 18 | 5.3 | 1.04 | 155 |
| 150 | 1 | 63 | -1.13 | 2.19 | 70 | 3.5 | 0.92 | 56 |
| 151 | 1 | 445 | 0.71 | 2.35 | 29 | 3.8 | 0.97 | 157 |
| Some values are missing and depicted as: “-“ ,  * Normal values are dependent on age and gender, hence the use of Z-scores based on own reference values in individuals aged 7-80 years old. | | | | | | | | |
